# Case report: Successful treatment of acute generalized pustular psoriasis of puerperium with secukinumab

**DOI:** 10.3389/fmed.2022.1072039

**Published:** 2022-12-08

**Authors:** Gao Xue, Ma Lili, Fang Yimiao, Wu Miao, Yang Xiaohong, Wang Dongmei

**Affiliations:** ^1^The First School of Clinical Medicine, Zhejiang Chinese Medical University, Hangzhou, China; ^2^The First Affiliated Hospital of Zhejiang Chinese Medical University (Zhejiang Provincial Hospital of Traditional Chinese Medicine), Hangzhou, China; ^3^Tongde Hospital of Zhejiang Province, Hangzhou, China

**Keywords:** GPP, AGEP, secukinumab, interleukin-17A inhibition, biologic therapy

## Abstract

Generalized pustular psoriasis (GPP) is a rare and severe form of psoriasis presenting with erythematous, aseptic pustules. Common systemic symptoms include fever and myalgias. The presentation of GPP resembles acute generalized exanthematous pustulosis (AGEP). However, the treatment of these two pathologies differs. While AGEP is self-limiting and treated with topical corticosteroids and constrain of systemic steroids. GPP treatment avoids corticosteroid, choosing acitretin, methotrexate, and cyclosporine as first-line agents. In this case report, a 27-year-old female with a medical history of AGEP presented to the hospital with extensive erythema and pustules. Complete blood count acute phase reactant analysis revealed an elevated white blood cell count and C-reactive protein (CRP). Two histopathological examinations revealed psoriatic hyperplasia of the epidermis with keratosis, along with Kogoj and Munro micro abscesses above the spina layer. Lymphocytic and neutrophilic infiltrate was present in the superficial derma layer along with vasodilation. The patient was diagnosed with GPP according to pathological and clinical criteria. Treatment was initiated with secukinumab because of the patient’s failure to respond to systemic treatment with Acitretin, methotrexate, and cyclosporin. Following 2 weeks of therapy with 300 mg of secukinumab, the pustular lesions had resolved. This study indicates the potential efficacy of secukinumab as an effective therapy that can rapidly improve the clinical symptoms of GPP.

## Introduction

**FIGURE 1:**
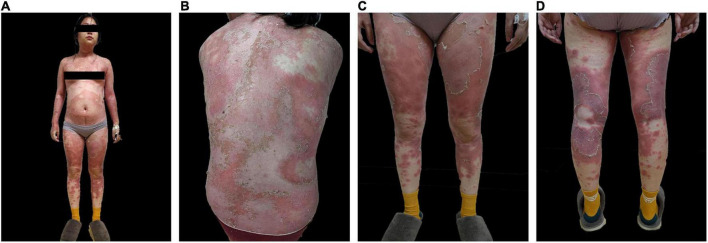
Pre-treatment skin images of **(A)** anterior skin lesions **(B)** posterior skin lesions **(C)** anterior lower extremities **(D)** posterior lower extremities.

**FIGURE 2:**
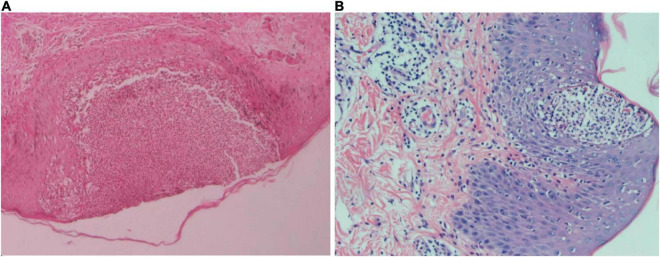
**(A)** Skin biopsy of the left lower limb with hematoxylin-eosin staining at 50× magnification. Squamous epithelial hyperplasia was observed with epidermal pustular formations. Kogoj abscesses can be seen at the pustular margin. The upper dermis showed lymphocytic infiltration and some dermal papillary edema. Perivascular infiltration and a small number of lymphocytes were also observed. **(B)** Skin biopsy of the left lumbar region with hematoxylin and eosin staining at 100× magnification. Squamous epithelial hyperplasia, Munro micro abscesses, and Kogoj abscesses were observed in the epidermis. A small amount of lymphocytic and neutrophilic infiltrate was observed in the upper dermis. Some dermal papillary edema, vascular dilation hyperemia and lymphocytic perivascular infiltration was also observed.

On the first of December 2020, a 27-year-old female presented to our hospital with systemic erythema and pustular lesions for 2 months. Two months prior, the patient had a successful cesarean delivery at a local hospital and given penicillin and oxytocin post-operatively. The patient reported the gradual development of erythema spreading from her torso to her extremities. Afterward, dense millet pustules appeared in addition to desquamation of the skin. Examination of the electronic medical record indicated that the patient had been hospitalized with acute generalized exanthematous pustulosis(AGEP) in an outside hospital. However, treatment was refractory to antibiotics and corticosteroid. At admission, multiple yellow-white scales and crusts involved 80% of the body surface area (BSA). The dermatology life quality index (DLQI) at admission was 27 with a static global assessment of psoriasis (PGA) of 4.7. The patient reported erythema of the forearms and trunk with dense, large military pustules, with areas of desquamation (Figure 1). Routine laboratory testing revealed leukocytosis with neutrophilic predominance, elevated C-reactive protein (CRP) as well as elevations in hepatic enzymes. Two histopathological examinations revealed psoriatic hyperplasia of the epidermis with keratosis with Kogoj and Munro micro abscess above the spina layer. Lymphocytic and neutrophilic infiltrate was also present in the superficial dermis (Figure 2). Following histopathological and clinical criteria, the patient received a diagnosis of generalized pustular psoriasis (GPP).

**FIGURE 3:**
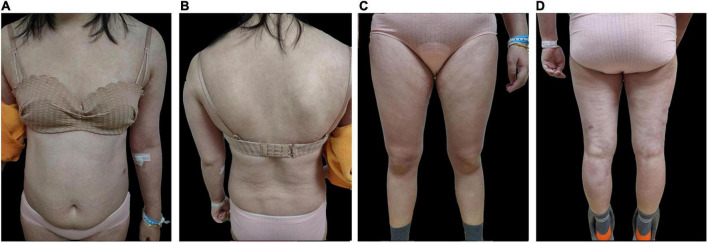
Post-treatment skin images of **(A)** anterior skin lesions **(B)** posterior skin lesions **(C)** anterior lower extremities **(D)** posterior lower extremities.

On admission, the patient was treated with intravenous injection of levofloxacin and compound glycyrrhizin, oral acitretin and thalidomide for 3 days, but no improvement was observed. On the contrary, he showed worse signs including continuous enlargement of erythema and pustule areas, superficial red erosion of the buccal mucosa, and targeted lesions of edematous erythema on both lower limbs. In addition, he reported muscle pain throughout his body, decreased appetite, and depression as well. Following Japanese guidelines on the treatment of refractory GPP, the patient was given 300 mg of secukinumab after being informed of the risks of treatment. Within 72 h, the erythema, pain, scales, and pustules were significantly relieved, and the patient’s mental state was improved (Figure 3). The DLQI decreased rapidly from 27 to 2 at week 3. After 5 injections once a week, maintenance therapy was initiated at a monthly basis for 3 months. A follow-up of 8 months showed no recurrence of skin lesions.

## Discussion

This case report details a misdiagnosis of AGEP and the inappropriate use of corticosteroid treatment in an outside hospital. As such, the patient’s condition worsened rapidly. Two epidermal biopsies showed psoriatic hyperplasia accompanied by incomplete keratosis, Kogoj microabscess, and dilatation and tortuosity of the superficial dermis. The histopathological features of AGEP include keratinocyte necrosis, dermal papillary edema, vasculitis, and perivascular eosinophil infiltration (1). GPP generally does not present with vasculitis and eosinophil infiltration. Therefore, the correct interpretation and clinical manifestations of early pathological diagnosis are of great significance for the treatment of GPP. This case reports the use of clinical judgment and histopathological analysis for the differential diagnosis between AGEP and GPP.

It has been reported that approximately 65% of GPP is secondary to psoriasis vulgaris (2). However, GPP also occurs in patients with no prior diagnosis of psoriasis, which can be induced by infection or in response to certain drugs. Specifically, prior studies have suggested that IV penicillin could induce GPP (3). The patient, who had no clear history of psoriasis, developed edematous erythema with pustular changes in his torso after intravenous penicillin infusion, which did not improve after discontinuation. Therefore, it is reasonable to conclude that GPP may have been induced by penicillin, which led to the early misdiagnosis of AGEP. This difference in treatment could have profound impacts for patients and should be assessed clinically prior to starting therapy.

First-line treatment for GPP currently includes acitretin, cyclosporine, methotrexate, or infliximab (4). In recent years, biologic agents targeting TNF-α, IL-1, IL-12/23p40, IL-17A, and IL-36 have been reported in the treatment of GPP (5, 6). Designed to target and neutralize IL-17A, secukinumab, a human monoclonal antibody, was evaluated for the treatment of GPP in a Phase III clinical study in Japan (7), and there are many real-world studies on pustular psoriasis in children (8, 9). Due to the patient’s lack of response to standard therapy, the patient was given secukinumab for a total of 3 months. After the initial dose, disease progression was halted and recovery of the lesions began promptly, with a resolution of pustules and erythema in 72 h. An improvement of the dermatology-specific health-related quality of life was also observed.

Currently, there are no unified guidelines for the long-term use of Secukinumab for the treatment of GPP in China. This patient received a weekly dose of 300 mg subcutaneously on week’s 0–4, followed by 300 mg every 4 weeks after (10). In this case, the lesions were completely cleared within 2 weeks of treatment with Secukinumab. The treatment duration was extended for a total of 3 months using the dosing schedule described above. At the 8 months follow up, no recurrence was reported. As such, Secukinumab may serve as a viable therapy for severe refractory cases of GPP.

## Data availability statement

The original contributions presented in this study are included in the article/supplementary material, further inquiries can be directed to the corresponding author.

## Ethics statement

Written informed consent was obtained from the individual(s) for the publication of any potentially identifiable images or data included in this article.

## Author contributions

GX collected the data of the case and finished the manuscript. ML provided guidance of modifying the manuscript. FY and WM helped collect the data of the case. WD helped polish the manuscript. All authors contributed to the article and approved the submitted version.
